# Croup Is One of the Clinical Manifestations of Novel Coronavirus in Children

**DOI:** 10.1155/2021/8877182

**Published:** 2021-03-02

**Authors:** Mojtaba Kamali Aghdam, Hosein Shabani Mirzaee, Kambiz Eftekhari

**Affiliations:** ^1^Pediatric Department, Mousavi Hospital, Zanjan University of Medical Sciences, Zanjan, Iran; ^2^Department of Pediatric, Bahrami Children's Hospital, Tehran University of Medical Sciences, Tehran, Iran; ^3^Pediatric Gastroenterology and Hepatology Research Centre, Department of Pediatric, Bahrami Children's Hospital, Tehran University of Medical Sciences, Tehran, Iran

## Abstract

The manifestations of novel coronavirus are diverse and can manifest through respiratory, gastrointestinal, and even nervous symptoms. Respiratory involvement is usually an upper tract infection or pneumonia but can also present as other forms of pulmonary disorders. A 3-year-old boy presented with cough, hoarseness, and stridor. He was treated with dexamethasone and nebulized adrenaline and a clinical diagnosis of croup was established. After treatment, his symptoms improved for a short time, but suddenly cough exacerbated and was accompanied by respiratory failure and seizures. He was then intubated and mechanically ventilated. Because of the coronavirus epidemic, Reverse-Transcription Polymerase Chain Reaction (RT-PCR) assay was taken from the pharyngeal secretions and was positive. The child was isolated. Due to excessive respiratory secretions and worsening of the general condition, bronchoscopy was performed depicting an image compatible with bacterial tracheitis. He was treated with broad-spectrum antibiotics, antivirals, and supportive care. Finally, after 4 weeks of treatment, the child was discharged in good general condition. Croup is one of the respiratory symptoms of novel coronavirus and can be a risk factor for bacterial tracheitis. Therefore, the presence of clinical manifestations of croup indicates the need for coronavirus PCR testing.

## 1. Introduction

The novel coronavirus first appeared in Wuhan, China. It quickly spread around the world and became known as COVID-19 [1]. The incidence of this infection in children younger than 18 years is relatively low (about 2.4% of all reported cases) [2]. The clinical manifestations in children are different from those in adults and are usually milder. The disease occurs in children secondary to home exposure to COVID-19 [1]. The main clinical presentation includes fever, chills, myalgia, weakness, and respiratory symptoms like cough, tachypnea, retraction, and shortness of breath [1]. Respiratory involvement is usually an upper respiratory tract infection or pneumonia but can also manifest as other forms of pulmonary disorders [3]. According to our knowledge, few cases of croup following COVID-19 have been reported in children, but no bacterial tracheitis has been reported following COVID-19 croup [4, 5]; only one case of bacterial superinfection pneumonia has been reported [5]. However, based on the research of the scientific literature, two cases of laryngotracheitis secondary to COVID-19 have been reported in adults [6].

Croup is a heterogeneous group of acute infectious processes characterized by bark-like cough that can be accompanied by hoarseness, expiratory stridor, and respiratory distress [7]. Bark-like cough is a hallmark of the disease in infants and young children, while hoarseness is predominant in older children and adults [8]. The causes of croup are viral infections. Approximately 75% of cases are parainfluenza virus, and the rest include influenza A and B, adenovirus, respiratory syncytial virus (RSV), and measles [7]. Human coronavirus NL63 (HCoV-NL63) is another cause of croup [8]. The frequency of complications in children with croup is 15% [7]. Bacterial tracheitis is one of the most serious complications of croup and is associated with the risk of airway obstruction [7, 8]. It usually follows a viral croup and is rarely seen initially [7]. The incidence of the disease is very rare in summer or spring [9].

## 2. Case Presentation

A 3-year-old boy was brought to the emergency department due to cough, hoarseness, and evident stridor. He was hospitalized with a diagnosis of croup and treated with nebulized adrenaline and dexamethasone. The condition improved after few hours and was then discharged. He is the third child of nonconsanguineous parents, without a history of any underlying disease. Soon after discharge, he returned due to exacerbation of cough, respiratory distress, generalized seizures, and an upwards gaze. The seizure lasted about ten minutes. There was no previous history of seizures in him and his relatives. At first, the child was fully conscious, but after a short time he developed severe cough, shortness of breath, and toxicity. He was then intubated and transferred to the Pediatric Intensive Care Unit (PICU). His treatment was started with vancomycin (45 mg/kg/day divided into 4 doses—made by Exir Pharmaceutical Co., Iran), meropenem (120 mg/kg/day divided into three doses—made by Dana Pharmaceutical Co., Iran), and phenytoin. The necessary paraclinical tests were sent. PCR test for coronavirus was taken from pharyngeal secretions because of the coronavirus epidemic and a history of attending a party a few days before admission. The child's family did not have symptoms of COVID-19 and all of them Coronavirus PCR tested negative. He had a history of being in a group of children, and the disease probably spreads from there. The results of the lab test were as follows:

WBC = 7 × 10^3^/*μ*L (L: 19%, N: 75%), (normal range: 4 − 10 × 10^3^/*μ*L), Hb = 11.3 g/dl, PLT = 168 × 10^3^/*μ*L (normal range: 150 − 450 × 10^6^/*μ*L), BS = 110 mg/dl (normal range: 50-115 mg/dl), BUN = 16 mg/dl (normal range: 5-18 mg/dl), Cr = 0.4 mg/dl (normal range: 0.3-0.7 mg/dl), Ca = 8.5 mg/dl (normal range: 8-13 mg/dl), Na = 140 mEq/L (normal range: 136-146 mEq/L), K = 4.5 mEq/L (normal range: 3.5-5.1 mEq/L), CRP = 48 mg/dl (normal range: < 5 negative), AST = 40 U/L (normal range: up to 41 U/L), ALT = 22 (normal range: up to 41 U/L), PT = 12 seconds (normal range: 11-13.5 seconds), INR = 1 (normal range: 0.8-1.1), PTT = 30 seconds (normal range: 25-35 seconds), LDH = 1677 U/L (normal range: 230-460 U/L), ESR = 30 mm/hr (normal range: < 10 mm/hr), blood culture = negative.

**Figure 1:**
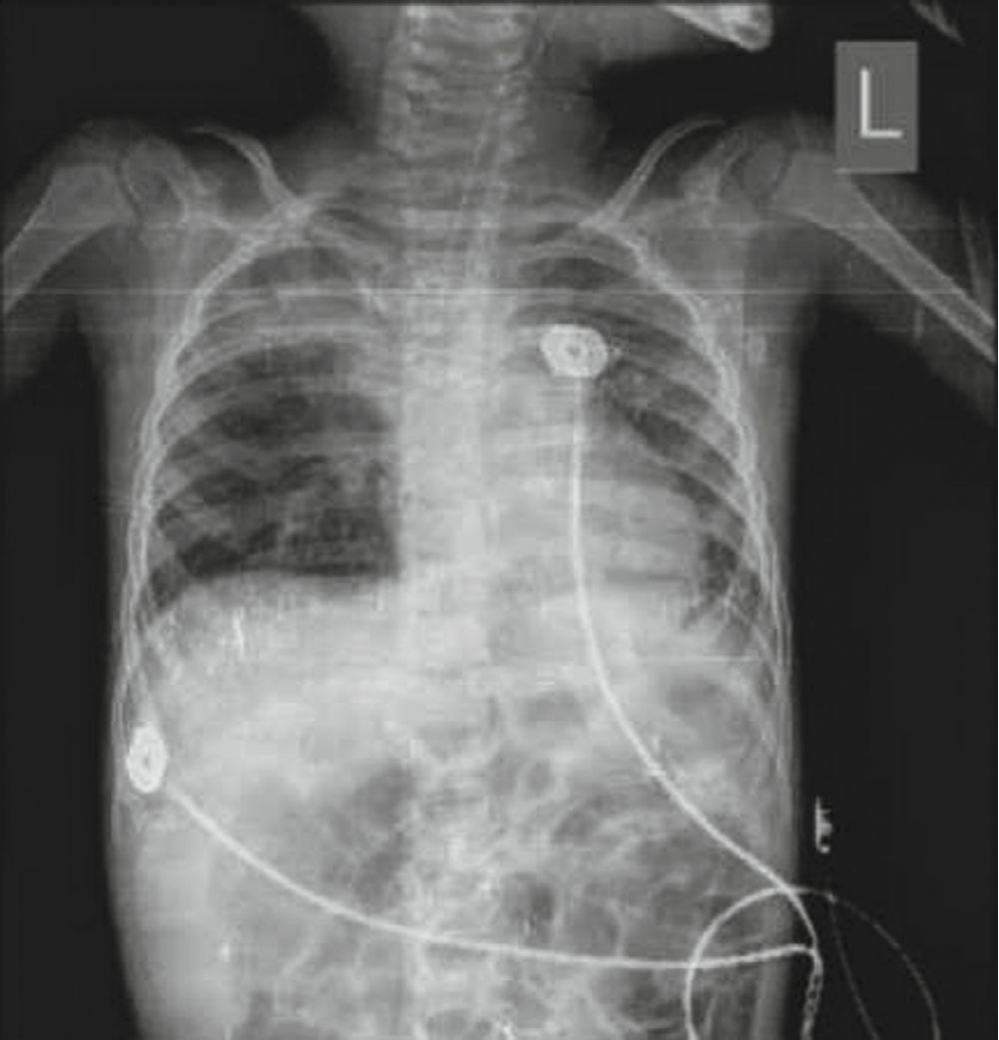
Chest X-ray of the patient.

Contact isolation was initiated, but his condition gradually worsened, as stridor and respiratory discharge increased. Chest X-ray showed bilateral peribronchial cuffing at the parahilar region and consolidation at the right upper lobe (Figure 1).

Bronchoscopy was performed quickly. A lot of thick and purulent discharge was found in the trachea and bronchi, suggesting bacterial tracheitis. A sample of this purulent discharge was sent for culture, which was positive for Staphylococcus aureus. Several generalized seizures occurred during hospitalization that were controlled with phenobarbital. Coronavirus PCR test was positive; therefore, although the treatment of coronavirus in children is mostly based on symptomatic, supportive, and complication treatment, Kaletra (Ritonavir/lopinavir) (made by HETERO Pharmaceutical Co., India) was started with a dose of 12 mg/kg/12 h (lopinavir component). The decision was taken according to the protocol of the Iran Ministry of Health. He was under mechanical ventilation for 17 days, receiving supportive care and parenteral nutrition. Gradually, his general condition improved and was finally discharged. In the follow-up, the patient was completely well without any complaint.

## 3. Discussion

Parainfluenza virus is the most common cause of croup (laryngotracheobronchitis) in children. Other viruses can also cause croup, including influenza A and B, adenovirus, RSV, measles, and HCoV-NL63 [7, 8]. Only a few cases of laryngotracheobronchitis have been reported in children following COVID-19 [4, 5]. Interestingly, in our patients, there were typical croup symptoms before the onset of bacterial tracheitis. Initially, he responded well to the conventional therapy of nebulized adrenaline and dexamethasone as described in Pitstick's study [4]. After the onset of bacterial tracheitis, the course of the disease worsened and required mechanical ventilation. In fact, bacterial tracheitis by Staphylococcus aureus is a superinfection that occurs when the organism is added to an underlying viral disease such as coronavirus. However, in the study of Venn et al., initially, patients with croup did not respond well to treatment but after several hours recovered [5]. Unlike most cases of croup, which is more common in the autumn and winter [9], our patient developed croup during summer as in Venn's study [5]. In the study of Pitstick et al., the disease was also reported in the spring [4]. In our patient, contrary to most reports, the child's relatives had no symptoms of infection, and the child probably got the disease from a pediatric group. In some cases, following croup secondary to COVID-19, bacterial airway infection may present requiring intubation and mechanical ventilation. In our case, this was due to bacterial tracheitis and in other cases pneumonia [5]. An important point in these patients was a complete recovery, even in very severe cases, and discharge in good general condition. Given the current coronavirus epidemic, and the result of this case report, it is recommended that screening of COVID-19 be performed on children with croup symptoms; on the other hand, respiratory and contact isolation should be done to prevent infection spread.

## 4. Conclusion

Croup can be one of the clinical manifestations of COVID-19 in children. This may be complicated by the bacterial superinfection and cause bacterial tracheitis.

## Data Availability

All relevant data have been included in the article.
